# The association of calcium intake with osteoporotic vertebral fractures in a large Chinese cohort

**DOI:** 10.18632/aging.102974

**Published:** 2020-03-28

**Authors:** Ling Wang, Lu Yin, Xiaoguang Cheng, Kai Li, Yuebo Wang, Yong Zhang, Yang-yang Duanmu, Xiaoyun Liu, Guijuan Deng, Yang Wang, Nicola Veronese, Wei Li, Wei Tian, PURE China CASH study team

**Affiliations:** 1 Department of Radiology, Beijing Jishuitan Hospital, Beijing 100035, China; 2 Medical Research and Biometrics Center, National Center for Cardiovascular Disease, Beijing 102300, China; 3 Aging Branch (N.V.), National Research Council, Neuroscience Institute, Padova, Italy; 4 Department of Spine Surgery, Beijing Jishuitan Hospital, Beijing 100035, China

**Keywords:** calcium intake, vertebral fracture, QCT

## Abstract

The effect of calcium on prevention of osteoporosis and related fracture which are aging issues is unclear. The aim of this study is to explore the association of calcium intake with vertebral fracture. This study enrolled 3,457 participants from China Action on Spine and Hip Status (CASH) study from 2013 and 2017. Dietary calcium intake was collected using validated food frequency questionnaires (FFQ). Vertebral fracture of CT images was defined as the primary outcome. The mean calcium intake of men and women were 522.75mg/day and 507.21mg/day, respectively. 6% reduction in the odds of fracture risk was observed per 100 unit increase of calcium intake from food among females (OR, 0.94; 95% CI, 0.89-0.99), but results among males were not significant. We divided calcium intake into quintiles when modelling its associations with fracture risk, negative associations of fracture risk with calcium intake were found among females. In a population with low usual calcium intake, higher dietary calcium intake was associated with fewer vertebral fracture in women and that no such association was seen in men.

## INTRODUCTION

Osteoporotic fractures are a global public health challenge associated with aging, and commonly occur in the spine, hip, forearm and other skeletal sites [1]. Vertebral fractures can cause serious morbidity and excess mortality, including chronic pain and disabilities, dependence increase [2]. Calcium is widely recognized as an effective intervention for the prevention of osteoporosis, and older people are recommended to take at least 1000-1200 mg/day of calcium to treat and prevent osteoporosis [3]. However, some recent studies and meta-analyses indicate that calcium supplements may be ineffective to prevent fracture [4–7].

Other studies have raised concerns about the safety of calcium supplements, including cardiovascular events, urinary tract stones, gastrointestinal symptoms, and hospital admissions for acute gastrointestinal problems [7–10]. These reports have led to suggestions that calcium intake should be increased through food rather than by taking supplements, although the effect of increasing dietary calcium intake on bone health is uncertain [11]. Furthermore, because most studies were conducted in Caucasian populations with moderate to high dietary calcium intake, little is known about the association between dietary calcium intake and fractures in populations with low calcium intake. The mean dietary calcium intake is low in China (300-400 mg/d), in Korea (300–500 mg/d) and in Japan (400–500 mg/d) [12–14]. These values are much lower than those reported in Western populations (700–1300 mg/d) [15]. The dose interaction between calcium intake and bone health may differ according to baseline dietary habits and/or ethnicity [6]. Whether calcium intake across the typical dietary range influences the preservation of bone mass has not been established in Asians.

Most previous studies including meta-analyses evaluated actual fracture requiring professional treatments or self-reported fracture as endpoints, so fracture incidence and/or prevalence were around 2-7% within 5 years. In this article, we report data for subjects enrolled in the China Action on Spine and Hip (CASH) study China cohort with low dietary calcium intake at baseline, who had spinal quantitative computed tomography (QCT) volumetric bone mineral density (vBMD) measurements and vertebral fracture assessment at their median of 10 years visit to examine whether calcium intake in food is associated with vertebral fracture.

## RESULTS

**Figure 1:**
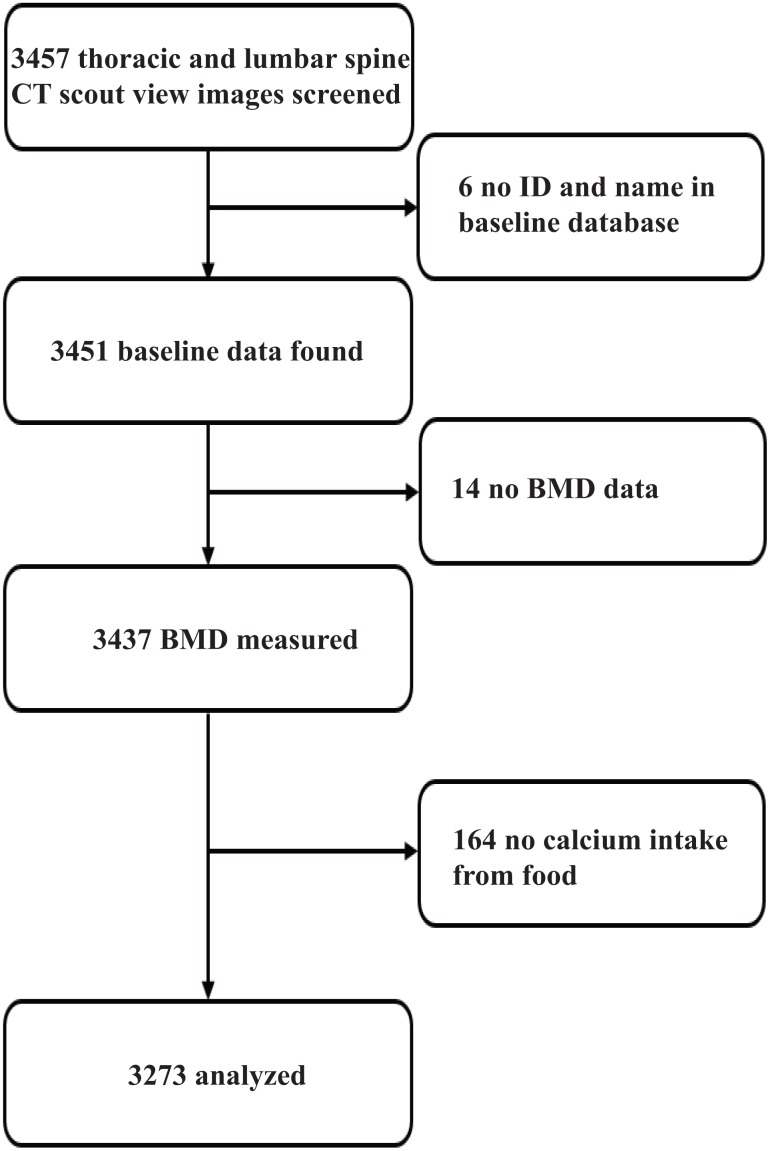
**Participant selection for final analyses.**

Of the 3457 CASH participants, 6 were excluded because their ID could not be found in the baseline database. For the analysis of the associations of calcium intake with fracture and BMD, a further 14 were removed due to missing BMD results, and 164 excluded due to missing data on calcium intake. Finally, 3273 (94.7%) were used for analysis (Figure 1).

The self-reported vertebrae fracture prevalence of this cohort at baseline is 3.56% (Supplementary Figure 1A). 82.73% of fracture occurred with only one site (Supplementary Figure 1B). Interestingly, the prevalence of fracture or vertebra fracture at baseline was higher in high calcium intake quintile groups in both sexes (Table 1). Total vertebral fracture prevalence was 16.5% (n=551). The most common location was at the 12^th^ thoracic vertebra (TV) (6.11%, n=206), next the 1^st^ lumbar vertebra (LV) (5.75%, n=188), and the thirdly the 11^th^ TV (4.73%, n=154) (Supplementary Figure 2A). 76.0% of thoracic fracture occurred with only one vertebrae, while 82.5% of lumber fracture occurred with one site (Supplementary Figure 2B). Among the 3273 participants, 62.7% were females and the mean age of the fracture groups was older than those without fracture (P<0.01). The prevalence of mild vertebral fracture was 11.2% and 15.7% among females and males, respectively, and 4.6% and 2.9% for moderate or severe fracture. Fracture prevalence was much higher among older adults than younger adults of age≤60 years. The mean daily calcium intake from the FFQ was imputed as 517.0±266.4 mg. The mean calcium intake of men and women were 522.75mg/day and 507.21mg/day, respectively. Detailed results of the associations of fracture risk with each potential factor among females and males are presented in Table 1 by quintiles of calcium intake from food. 6% reduction in the odds of fracture risk was observed per 100 unit increase of calcium intake from food among females (OR, 0.94; 95% CI, 0.89-0.99), but results among males were not significant (OR, 0.99; 95% CI, 0.93-1.05). We divided calcium intake into quintiles when modelling its associations with fracture risk, negative associations of fracture risk with calcium intake were found among females (Q4 vs. Q1: OR, 0.54; 95% CI, 0.36-0.82; Q5 vs. Q1: OR, 0.55; 95% CI, 0.37-0.83), but this trend was not shown for lower calcium intake quintile groups (Q2 vs. Q1: OR, 0.78; 95% CI, 0.53-1.16; Q3 vs. Q1: OR, 0.74; 95% CI, 0.50-1.09). The same regressions were performed for male participants, but no significance was found, whatever for unadjusted and adjusted models (Table 2). Table 3 shows 6% reduction in the odds of fracture risk associated with per 100 unit increase of calcium intake from food among females in urban region (OR, 0.94; 95% CI, 0.88-1.00), however, no significant outcomes were observed among females in rural areas or among men in urban or rural regions. Supplementary Tables 2 and 3 demonstrate sensitivity analyses based on menopausal status at baseline and age groups (≥ 55 years as a surrogate for post-menopause [16], the 95^th^ percentile of the age of menopause in PURE-China).

**Table 1:** The characteristics of subjects participating by quintiles of calcium intake from food.

**Characteristics**	**Calcium quintiles (mg/day)**					**P value**
	**Q1 (≤296)**	**Q2 (297-395)**	**Q3 (396-519)**	**Q4 (520-706)**	**Q5 (>706)**	
**Females, N**	**411**	**405**	**390**	**409**	**437**	
Age at spine examination (years)	60.3±9.0	60.6±9.2	62.1±9.1	62.9±9.0	61.1±8.9	<0.01
Age>60 years (%)	54.3	52.6	58.7	61.4	56.3	0.09
College or higher (%)	1.0	5.0	3.6	11.3	14.0	<0.01
Living in rural areas (%)	78.6	60.2	46.7	26.4	14.2	<0.01
BMI (kg/m^2^)	24.1±3.9	24.4±3.4	24.2±3.4	24.7±3.7	24.4±3.6	0.19
BMI≥25 kg/m^2^ (%)	34.6	40.4	37.7	41.6	40.3	0.24
Waist (cm)	77.8±9.5	78.7±9.7	78.7±8.9	79.1±9.8	77.8±9.6	0.30
Waist>89 cm (%)	11.9	15.6	12.6	15.2	12.4	0.38
Current or ex-smokers (%)	4.9	4.7	2.4	2.0	1.8	0.01
Current or ex-drinkers (%)	3.9	5.2	6.4	4.7	7.8	0.11
Self-reported fracture at baseline (%)	7.3	6.7	9.2	10.5	11.7	<0.01
Self-reported vertebral fracture at baseline (%)	2.4	2.7	2.8	4.9	4.4	0.03
Calcium intake (mg/day)	235.9±41.3	344.6±29	452.5±35.5	602.6±51.9	945.5±227	<0.01
BMD (mg/cm^3^)	98.4±40.6	99.2±40.7	98.8±43.4	95.7±38.9	104.2±41.3	0.04
Osteopenia (%)	33.6	36.3	34.1	40.1	39.6	0.24
Osteoporosis (%)	37.2	35.8	38.0	35.7	27.0	0.01
Levels of physical activities						<0.01
<600 minutes/week	19.3	13.3	8.4	7.2	5.2	
600-3000 minutes/week	40.1	40.7	42.4	52.1	44.7	
>3000 minutes/week	40.6	46.0	49.2	40.7	50.1	
Vertebral fracture (%)	19.5	16.3	17.4	14.2	11.9	0.03
Mild vertebral fracture (%)	13.4	11.1	12.0	10.5	9.2	0.27
Moderate/sever vertebral fracture (%)	6.1	5.2	5.4	3.7	2.8	0.09
**Males, N**	**242**	**248**	**267**	**246**	**218**	
Age at spine examination (years)	61.0±9.6	61.5±8.6	62.9±9.3	63.9±9	63.7±9	<0.01
Age>60 years (%)	56.2	60.1	63.3	67.9	66.5	0.05
College or higher (%)	3.3	6.1	10.2	13.1	15.6	<0.01
Living in rural areas (%)	82.6	63.7	55.4	34.6	17.0	<0.01
BMI (kg/m^2^)	23.4±4.3	23.8±3.3	24.4±3.8	24.4±3.9	25.0±3.4	<0.01
BMI≥25 kg/m^2^ (%)	28.2	33.1	40.8	40.8	50.7	<0.01
Waist (cm)	79.5±10.0	81.3±10.1	83.4±9.5	83.4±10.2	85.6±10.0	<0.01
Waist>101 cm (%)	2.9	1.6	3.4	3.3	4.2	0.60
Current or ex-smokers (%)	52.7	55.6	56.1	45.9	50.5	0.14
Current or ex-drinkers (%)	46.1	48.0	52.8	49.6	50.5	0.62
Self-reported fracture at baseline (%)	5.8	7.7	9.0	11.0	9.6	0.05
Self-reported vertebral fracture at baseline (%)	2.1	2.0	3.4	4.9	4.6	0.03
Calcium intake (mg/day)	245.2±39.8	345.6±27.8	452.1±35.8	606.6±51.3	937.3±207.7	<0.01
BMD (mg/cm^3^)	116.8±36.5	111.2±34.9	112.4±36.7	109.2±33.5	107.9±35.4	0.08
Osteopenia (%)	40.9	39.5	37.8	39.0	47.2	0.18
Osteoporosis (%)	14.0	18.2	19.8	20.3	18.8	0.27
Levels of physical activities						0.02
<600 minutes/week	22.8	14.5	13.7	13.4	9.6	
600-3000 minutes/week	33.2	39.7	41.4	39.5	41.6	
>3000 minutes/week	44.0	45.7	44.9	47.1	48.8	
Vertebral fracture (%)	19.8	17.3	19.8	19.9	15.6	0.67
Mild vertebral fracture (%)	17.4	13.3	17.2	16.3	14.2	0.66
Moderate/sever vertebral fracture (%)	2.5	4.0	2.6	3.7	1.4	0.47

Note: BMI, body mass index; BMD, bone mineral density.

**Table 2:** The unadjusted and adjusted associations of vertebral fracture with calcium intake by gender.

**Calcium intake from food**	**Fracture risk % (n)**				**Dichotomous odds ratio (95% confidence interval)^1^**				**Ordinal odds ratio (95% confidence interval)^2^**		
	**No**	**Mild**	**Moderate or severe**		**Crude**	**Adjusted^3^**	**Adjusted^4^**		**Crude**	**Adjusted^3^**	**Adjusted^4^**
**Females**											
Per 100-unit increase	1728 (84.2)	230 (11.2)	94 (4.6)		0.94 (0.89, 0.98)	0.92 (0.87, 0.97)	0.94 (0.89, 0.99)		0.94 (0.89, 0.98)	0.92 (0.87, 0.96)	0.93 (0.88, 0.98)
*P value*					*0.01*	*<0.01*	0.02		0.01	*<0.01*	0.01
Calcium intake quintiles										
Q1	331 (80.5)	55 (13.4)	25 (6.1)		ref.	ref.	ref.		ref.	ref.	ref.
Q2	339 (83.7)	45 (11.1)	21 (5.2)		0.81 (0.56, 1.15)	0.77 (0.53, 1.12)	0.78 (0.53, 1.16)		0.81 (0.56, 1.15)	0.75 (0.51, 1.09)	0.76 (0.51, 1.11)
Q3	322 (82.6)	47 (12.0)	21 (5.4)		0.87 (0.61, 1.25)	0.72 (0.49, 1.05)	0.74 (0.50, 1.09)		0.87 (0.61, 1.25)	0.70 (0.48, 1.02)	0.72 (0.49, 1.06)
Q4	351 (85.8)	43 (10.5)	15 (3.7)		0.68 (0.47, 0.99)	0.51 (0.35, 0.76)	0.54 (0.36, 0.82)		0.68 (0.47, 0.98)	0.49 (0.33, 0.72)	0.52 (0.35, 0.77)
Q5	385 (88.1)	40 (9.2)	12 (2.8)		0.56 (0.38, 0.82)	0.49 (0.33, 0.73)	0.55 (0.37, 0.83)		0.55 (0.38, 0.81)	0.47 (0.32, 0.70)	0.52 (0.35, 0.79)
*P_trend_*					*0.03*	*0.02*	*0.02*		*0.02*	*<0.01*	*0.01*
**Males**											
Per 100-unit increase	994 (81.4)	192 (15.7)	35 (2.9)		0.98 (0.93, 1.04)	0.97 (0.92, 1.03)	0.99 (0.93, 1.05)		0.98 (0.92, 1.04)	0.97 (0.92, 1.03)	0.99 (0.93, 1.05)
*P value*					*0.51*	*0.37*	*0.67*		*0.48*	*0.35*	*0.66*
Calcium intake quintiles										
Q1	194 (80.2)	42 (17.4)	6 (2.5)		ref.	ref.	ref.		ref.	ref.	ref.
Q2	205 (82.7)	33 (13.3)	10 (4.0)		0.85 (0.54, 1.34)	0.85 (0.54, 1.34)	0.92 (0.57, 1.47)		0.87 (0.55, 1.37)	0.87 (0.55, 1.37)	0.95 (0.60, 1.52)
Q3	214 (80.2)	46 (17.2)	7 (2.6)		1.00 (0.65, 1.55)	0.96 (0.62, 1.49)	1.00 (0.63, 1.59)		1.00 (0.65, 1.55)	0.97 (0.62, 1.50)	1.01 (0.64, 1.60)
Q4	197 (80.1)	40 (16.3)	9 (3.7)		1.01 (0.64, 1.57)	0.95 (0.61, 1.48)	1.00 (0.63, 1.61)		1.02 (0.66, 1.59)	0.96 (0.62, 1.51)	1.02 (0.64, 1.64)
Q5	184 (84.4)	31 (14.2)	3 (1.4)		0.75 (0.46, 1.21)	0.71 (0.43, 1.15)	0.81 (0.48, 1.36)		0.74 (0.46, 1.21)	0.70 (0.43, 1.14)	0.81 (0.49, 1.36)
*P_trend_*					*0.67*	*0.64*	*0.91*		*0.67*	*0.63*	*0.91*

Note: Per 100-unit refers to per 100 mg.

^1^Mild, moderate, severe fracture were combined defined as fracture prevalent and no fracture.

^2^Three categories were defined as moderate/severe fracture, mild fracture, and no fracture.

^3^Adjusted for age.

^4^Adjusted for age, education level, BMI, waist circumference, tobacco use, alcohol consumption, and physical activities.

**Table 3:** The unadjusted and adjusted associations of vertebral fracture with calcium intake by gender and living location.

**Calcium intake from food**	**Fracture risk % (n)**				**Dichotomous odds ratio (95% confidence interval)^1^**				**Ordinal odds ratio (95% confidence interval)^2^**		
	**No**	**Mild**	**Moderate or severe**		**Crude**	**Adjusted^3^**	**Adjusted^4^**		**Crude**	**Adjusted^3^**	**Adjusted^4^**
Females in rural areas											
Per 100-unit increase	763 (83.0)	111 (12.1)	45 (4.9)		0.95 (0.86, 1.05)	0.98 (0.88, 1.09)	1.00 (0.90, 1.11)		0.95 (0.86, 1.04)	0.97 (0.87, 1.08)	0.99 (0.89, 1.10)
*P value*					*0.30*	*0.68*	*0.94*		*0.25*	*0.57*	*0.81*
Calcium intake quintiles											
Q1	260 (80.5)	47 (14.6)	16 (5.0)		ref.	ref.	ref.		ref.	ref.	ref.
Q2	205 (84.0)	27 (11.1)	12 (4.9)		0.80 (0.51, 1.23)	0.82 (0.52, 1.29)	0.84 (0.53, 1.35)		0.79 (0.51, 1.22)	0.82 (0.51, 1.30)	0.86 (0.54, 1.39)
Q3	151 (83.0)	22 (12.1)	9 (5.0)		0.86 (0.53, 1.37)	0.80 (0.49, 1.31)	0.85 (0.51, 1.42)		0.85 (0.53, 1.36)	0.79 (0.48, 1.31)	0.85 (0.50, 1.43)
Q4	91 (84.3)	12 (11.1)	5 (4.6)		0.78 (0.44, 1.40)	0.79 (0.43, 1.46)	0.78 (0.41, 1.49)		0.77 (0.43, 1.39)	0.79 (0.43, 1.47)	0.77 (0.40, 1.49)
Q5	56 (90.3)	3 (4.8)	3 (4.8)		0.46 (0.19, 1.10)	0.62 (0.25, 1.54)	0.71 (0.28, 1.78)		0.44 (0.18, 1.07)	0.57 (0.23, 1.46)	0.66 (0.26, 1.69)
*P_trend_*					*0.45*	*0.75*	*0.88*		*0.41*	*0.71*	*0.86*
Females in urban areas										
Per 100-unit increase	965 (85.2)	119 (10.5)	49 (4.3)		0.93 (0.88, 0.99)	0.96 (0.90, 1.03)	0.98 (0.91, 1.05)		0.94 (0.88, 1.00)	0.97 (0.91, 1.04)	0.98 (0.92, 1.05)
*P value*					*0.03*	*0.28*	*0.51*		*0.04*	*0.40*	*0.63*
Calcium intake quintiles										
Q1	71 (80.7)	8 (9.1)	9 (10.2)		ref.	ref.	ref.		ref.	ref.	ref.
Q2	134 (83.2)	18 (11.2)	9 (5.6)		0.80 (0.41, 1.55)	0.85 (0.43, 1.69)	0.84 (0.40, 1.77)		0.84 (0.43, 1.65)	0.94 (0.46, 1.90)	0.91 (0.42, 1.96)
Q3	171 (82.2)	25 (12.0)	12 (5.8)		0.86 (0.46, 1.61)	0.86 (0.45, 1.64)	0.92 (0.46, 1.86)		0.90 (0.48, 1.71)	0.94 (0.48, 1.85)	0.98 (0.47, 2.03)
Q4	260 (86.4)	31 (10.3)	10 (3.3)		0.62 (0.34, 1.14)	0.58 (0.31, 1.10)	0.66 (0.33, 1.32)		0.66 (0.35, 1.23)	0.66 (0.34, 1.27)	0.73 (0.36, 1.48)
Q5	329 (87.7)	37 (9.9)	9 (2.4)		0.55 (0.30, 1.00)	0.68 (0.36, 1.27)	0.77 (0.39, 1.51)		0.58 (0.32, 1.08)	0.77 (0.40, 1.47)	0.85 (0.42, 1.72)
*P_trend_*					*0.16*	*0.36*	*0.68*		*0.23*	*0.54*	*0.81*
Males in rural areas											
Per 100-unit increase	496 (79.0)	110 (17.5)	22 (3.5)		0.93 (0.83, 1.04)	0.94 (0.84, 1.05)	0.92 (0.81, 1.04)		0.93 (0.83, 1.04)	0.94 (0.84, 1.05)	0.92 (0.81, 1.03)
*P value*					*0.20*	*0.24*	*0.17*		*0.20*	*0.24*	*0.15*
Calcium intake quintiles											
Q1	155 (77.5)	39 (19.5)	6 (3.0)		ref.	ref.	ref.		ref.	ref.	ref.
Q2	125 (79.1)	24 (15.2)	9 (5.7)		0.95 (0.57, 1.56)	0.97 (0.59, 1.61)	1.00 (0.59, 1.69)		0.91 (0.55, 1.51)	0.93 (0.56, 1.55)	0.93 (0.55, 1.58)
Q3	112 (75.7)	31 (21.0)	5 (3.4)		1.11 (0.67, 1.83)	1.09 (0.66, 1.79)	1.07 (0.63, 1.82)		1.11 (0.67, 1.83)	1.08 (0.65, 1.79)	1.05 (0.62, 1.80)
Q4	71 (83.5)	13 (15.3)	1 (1.2)		0.68 (0.35, 1.31)	0.70 (0.36, 1.36)	0.65 (0.32, 1.34)		0.68 (0.35, 1.32)	0.70 (0.36, 1.36)	0.65 (0.32, 1.33)
Q5	33 (89.2)	3 (8.1)	1 (2.7)		0.43 (0.14, 1.26)	0.44 (0.15, 1.30)	0.35 (0.10, 1.19)		0.42 (0.14, 1.24)	0.43 (0.14, 1.27)	0.33 (0.10, 1.15)
*P_trend_*					*0.36*	*0.43*	*0.33*		*0.35*	*0.42*	*0.33*
Males in urban areas											
Per 100-unit increase	498 (84.0)	82 (13.8)	13 (2.2)		1.06 (0.98, 1.15)	1.06 (0.98, 1.15)	1.07 (0.98, 1.16)		1.06 (0.98, 1.15)	1.06 (0.98, 1.15)	1.07 (0.98, 1.16)
*P value*					*0.16*	*0.16*	*0.13*		*0.15*	*0.15*	*0.13*
Calcium intake quintiles											
Q1	39 (92.9)	3 (7.1)	0 (0.0)		ref.	ref.	ref.		ref.	ref.	ref.
Q2	80 (88.9)	9 (10.0)	1 (1.1)		1.64 (0.42, 6.30)	1.59 (0.41, 6.17)	2.36 (0.49, 11.45)		1.63 (0.42, 6.24)	1.58 (0.41, 6.10)	2.35 (0.49, 11.38)
Q3	102 (85.7)	15 (12.6)	2 (1.7)		2.18 (0.60, 7.89)	2.10 (0.58, 7.65)	3.22 (0.70, 14.85)		2.17 (0.60, 7.80)	2.08 (0.58, 7.55)	3.19 (0.70, 14.68)
Q4	126 (78.3)	27 (16.8)	8 (5.0)		3.73 (1.09, 12.85)	3.41 (0.98, 11.81)	4.81 (1.09, 21.32)		3.61 (1.05, 12.38)	3.28 (0.95, 11.33)	4.66 (1.05, 20.59)
Q5	151 (83.4)	28 (15.5)	2 (1.1)		2.57 (0.74, 8.91)	2.46 (0.71, 8.57)	3.72 (0.83, 16.63)		2.58 (0.75, 8.90)	2.47 (0.71, 8.55)	3.74 (0.84, 16.66)
*P_trend_*					*0.07*	*0.13*	*0.14*		*0.09*	*0.16*	*0.16*

^1^Mild, moderate, severe fracture were combined defined as fracture prevalent and no fracture (ref.).

^2^Three categories were defined as moderate/severe fracture, mild fracture, and no fracture (ref.).

^3^Adjusted for age.

^4^Adjusted for age, education level, BMI, waist circumference, tobacco use, alcohol consumption, and physical activities.

**Figure 2:**
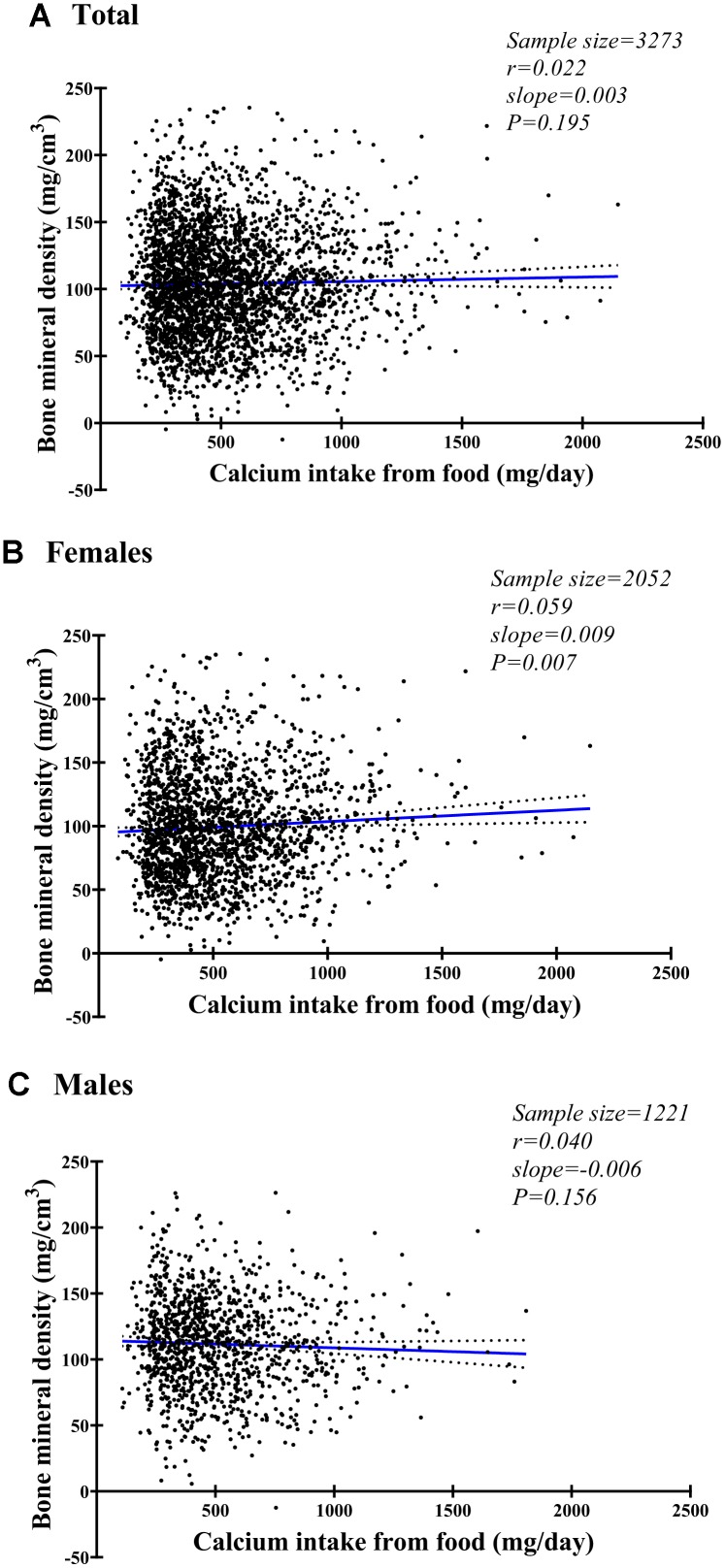
Fitting plot for spinal bone mineral density and daily calcium intake from food among females (**A**), males (**B**), and both (**C**).

Figure 2 shows the correlations between BMD and calcium intake in females, males, and both sexes combined. A positive correlation was observed for females (Figure 2B, P=0.01), but there was a non-significant negative trend for males (Figure 2C, P=0.16) and a non-significant positive trend for both sexes combined (Figure 2A, P=0.20).

## DISCUSSION

In this well-defined Chinese cohort with low usual dietary calcium intake, significant reduction of fracture risk was observed with increase of calcium intake from food among females, but not in males. Furthermore, negative associations of fracture risk with calcium intake were found among females. The present study provides significant evidence to support the hypothesis that higher calcium intake may prevent vertebral fractures for people with low usual calcium intake. This finding is inconsistent with several recent large randomized controlled trials of calcium supplements that failed to show any statistically significant evidence for fracture prevention [4, 17, 18]. Further, recent meta-analyses have failed to confirm any benefit from the use of calcium supplements in fracture prevention. However, most studies and meta-analysis including western population with high calcium intake, and the different calcium intake among the different populations may be an important confounding factor in interpreting the results of the studies on the effect of calcium on bone [19].

Several large cohort studies of calcium intake have used hip or total fracture incidence as the endpoint. Since hip fracture is the most serious consequence of osteoporosis and is associated with high mortality and morbidity [20], most studies of calcium intake have used this as their endpoint. However, little is known about the relationship for vertebral fractures, despite the fact that they are more common than hip fractures. Furthermore, vertebral fractures are often asymptomatic, so the incidence of self-reported vertebral fractures is often inaccurate, and a radiological examination is required for confirmation. The prevalence of radiographic vertebral fractures in China increases from 13% between ages 50 and 59 to over 50% after age 80 years [21]. A CT scout view image can be obtained with low radiation dose, and can be used to detect vertebral fracture [22, 23] with good agreement with a conventional X-ray [24].

Our results demonstrated higher dietary calcium intake associated with reduction of vertebral fracture in Chinese women. 6% reduction in vertebral fracture in women showed in the present study would be of great value in the large osteopenia/osteoporosis population. The recommended dietary calcium intake for elderly people is more than 1000 mg/day, yet the mean calcium intake in China of less than 400 mg/day [15]. Most studies finding no relation between dietary calcium intake and fracture incidence are based on populations with high calcium intake. Only one trial in frail elderly women in residential care with low dietary calcium intake and vitamin D concentrations showed a significant reduction in fracture risk [6]. While this might support the hypothesis that increasing calcium intake could be beneficial for those cohorts with low calcium intake, it is notable that populations in Asia have fewer fractures on calcium intakes of about 400 mg/day [15], and European and North American populations with high intake of dairy food and meat suffer from high fracture incidence. Recently in a large Korean community-based prospective study, Kong et al. did not find any association between calcium intake in food and fracture [25]. Conversely our results confirm the positive association of dietary calcium intake with preventing vertebral fracture in healthy older women. The discrepant outcomes might be caused by ages and different definitions of fracture (vertebral fracture diagnosed by scout view images, compared with any self-reported incident fracture in the Korean cohort study). The notable implication of our results for other societies is that the hypothesis of increasing dietary calcium intake being beneficial for individuals with dietary “calcium deficiency” should be more marked. Interestingly, our results show that the prevalence of fracture or vertebra fracture at baseline was higher in high calcium intake quintile groups in both sexes (Table 1). This may be due to participants who had fracture previously are more likely to increase their calcium intake, which means these results may reflect reverse causation.

Although the evidence of calcium intake reducing the prevalence of vertebral fractures is insufficient, many studies have demonstrated a beneficial effect of calcium intake on bone mineral density. Interestingly, we observed a small but significant positive correlation between calcium intake and vertebral vBMD in women. In a controlled clinical trial of the effect of calcium supplementation on bone density in older postmenopausal women, increasing daily calcium intake reduced bone loss in women with low calcium intake [26]. In another randomized controlled trial in a large sample of postmenopausal women, there is evidence calcium supplementation reduces bone turnover and it is associated with reduction in bone loss [27].

For Asian populations with low calcium intake, relevant data is little and the benefits of calcium on bone loss is unclear. A recent Japanese study showed that even a low-dose calcium supplementation (500 mg/day) was effective in preventing postmenopausal bone loss in the lumbar spine [28]. Another study from Hong Kong indicated that supplementing the diet of high calcium intake retards bone loss [29]. However, the sample sizes of the two above studies are small which limits their conclusions to be reached with great certainty. In another Korean national population study with mean daily calcium intake 470 mg/d, BMD in the lumbar spine (both sexes) and femoral neck (women) was significantly lower only when calcium intake was less than 400 mg/d [30]. What’s more, in men, femoral neck and total hip BMD was positively related to calcium intake up to 1200 mg/d [30]. Overall, our results suggest that higher calcium intake may provide substantial beneficial effects for BMD and supplementation in this population might have potential beneficial effects on prevention of fractures.

The potential causes for gender differences observed in this study are unclear. However, given the gender differences in fracture risk and calcium intake, it is plausible that different associations exist between women and men. Another important note is that our study with a sample size of 1221 male subjects may not be powered to assess the association of calcium intake with fracture risk and bone loss.

Our study strengths include the population based prospective design in both sexes in a setting with delegate imaging protocols. The vertebral fracture was assessed with lateral images of spine, and BMD was measured with the QCT, the most sensitive BMD measurement at present. Using repeat measurements of dietary intake in the sub-PURE China Cohort increased the accuracy and precision of measurements on dietary calcium intake. We performed a long follow-up time during which potential benefits of calcium intake could be shown despite of so low fracture incidence. We adjusted for several important covariates, although residual confounding may not be excluded. This study has some potential limitations. First, dietary calcium intake was measured only at baseline; therefore individual variations in calcium intake and diet during the follow-up period cannot be considered. Second, our calcium was imputed only from FFQ. Calcium supplements were not collected, though calcium supplements may be low in Chinese population [31]. Third, no data were available regarding serum biochemical indices such as bone turnover markers, and serum calcium and vitamin D that might explain the potential mechanisms of the effect of calcium on fracture. Finally, there are no CT scout views and no data on incident fractures at baseline.

In summary, in a population with low usual calcium intake, more calcium in the diet was associated with fewer vertebral fracture in women and that no such association was seen in men.

## MATERIALS AND METHODS

### Study design and participants

China Action on Spine and Hip Status (CASH) study (NCT 01758770) is a multi-center, community-based cohort study conducted by a research team from Beijing Jishuitan Hospital of Peking University, China [32]. The participants of CASH study were recruited from an ongoing community-based cohort study which were detailed in previous publications [33, 34], and spine and/or hip QCT and/or Dual-energy X-ray absorptiometry (DXA) were used to investigate the prevalence of osteoporosis and osteoarthritis in middle-age and older Chinese population. These participants aged 35-70 years old were recruited during 2005-2009, with mean age of 51.4±9.6 years [34]. Before bone examination, we required to confirm the availability of a CT scanner with CT centers located near the participant living areas and their willingness to provide a free CT scan for collaboration. Finally, 12 centers joined our sub-study during 2013-2017 (3 from Sichuan, 3 from Jiangsu, 2 from Beijing, 1 from Shanxi, 1 from Shaanxi, 1 from Liaoning, 1 from Jiangxi), and participants living close to any one CT center were invited. At the completion of this study, 3,457 participants from 12 centers underwent lumbar spine QCT scans with thoracic and lumbar spine CT scout views. The protocol and informed consent for the CASH study were reviewed and approved by the institutional review board of Beijing Jishuitan Hospital (approval number No. 201210-01; No. 201512-02).

### Data collection

The baseline data were collected with a median of 10 years before the spine CT screening of CASH study. Socio-demographic, tobacco use, alcohol consumption, and physical activities were collected via a structured questionnaire, and physical examination was conducted to collect weight, height, and waist circumference for each participant by trained physicians. In addition, detailed information on physical activity was collected using the International Physical Activity Questionnaire (IPAQ) [35]. A semi-quantitative Chinese Food Frequency Questionnaire (FFQ) with 149 items in 17 food categories was used to estimate average eating frequency and quantity in the past one year, which has been applied in several other studies including the Chinese National Nutrition and Health Survey in 2002 with satisfactory outcomes of reproducibility and validity [36]. Calcium from various foods were computed using the Chinese Food Composition Table database, and a 1.5% difference was reported in the intake of calcium between two FFQ [36, 37]. Chinese Dietary Reference Intakes (CDRI) recommended that Chinese adults should consume 800 mg calcium per day. All data mentioned above were derived from a baseline database to assess the association of calcium intake and vertebral fracture and spinal vBMD.

### QCT Volumetric BMD (vBMD) and vertebral fracture assessment protocol

All CT scans were performed at around 6-to-12-year follow-ups between March 2013 and August 2017. Details of the CT scanners at each center and the scanning parameters are given in Supplementary Table 1. For QCT, Mindways (Austin, TX, USA) QCT phantom and software were used at all centers. A CT scout view covering T4-S1 was obtained during the CT exam. For the upper abdomen, a regular CT scan was obtained with the predefined scan parameters and table height. A single European spine phantom (ESP, No.145) was circulated to each CT center for cross-calibration. All CT raw data and QCT data were transferred to the Radiology Department at the Beijing Jishuitan Hospital, which served as the quality control and data managing center for further analysis.

The volumetric bone mineral density (vBMD, mg/cm^3^) of the L1 and L2 vertebral bodies was measured using Mindways QCT pro v5.0 software according to the manufacturer’s recommendations. The average vBMD of L1-2 was taken as the average lumbar spine vBMD of each subject. The classification of osteoporosis using QCT vBMD was based on the International Society for Clinical Densitometry (ISCD) 2007 criteria [38], i.e., spine BMD 1) <80mg/cm^3^, osteoporosis, 2) 80-119 mg/cm^3^, osteopenia, and 3) ≥120 mg/cm^3^, normal.

The lateral CT scout view image was used to assess for vertebral fracture according to Genant’s semiquantitative (SQ) method [2, 39]. The SQ diagnostic approach as described by Genant et al for spine radiographs was used to detect vertebral body fractures on the scout view image of CT scan [39]. Each vertebral body was classified as normal (grade 0), mild (grade 1), moderate (grade 2) or severe (grade 3) fracture [39]. The digital images were displayed and viewed with a professional DICOM view work station and the reading was done by an expert MSK radiologist (CXG) with many years’ experience of vertebral fracture assessment. The subject was considered to have a vertebral osteoporotic fracture if any one of the T4-L4 vertebral bodies had a VFA score ≥grade 1. The highest VFA score in each individual was considered the fracture severity for that subject.

### Outcomes

The primary outcome was defined as vertebral fracture based on CT images and the main measures included volumetric bone mineral density at participants’ median of 10 years visit and dietary calcium intake at baseline.

### Statistical analyses

The subjects’ characteristics recorded at baseline were used to evaluate their associations with vertebral fractures. Continuous variables were shown as the mean ± standard deviation (SD), and categorical variables as numbers (n) and percentages (%). Kruskal-Wallis tests or chi-square tests were used for continuous variables or categorical variables among various fracture groups.

Gender-specific results were presented and analyzed by calcium intake quintiles of overall study population. Odds ratio (OR) and 95% confidence interval (CI) were obtained for the associations of dietary calcium with vertebral fracture from ordinal logistic regression models (if outcome defined as three categories, mild, moderate or severe) or dichotomous logistic regression models (when mild, moderate, or severe fracture were combined and defined as prevalent fracture and no fracture as reference group). Potential covariates including age, education level, BMI, waist circumference, tobacco use, alcohol consumption, and physical activities for multivariate regression models were selected based on previous literatures and univariate model analyses. The interaction between vertebral fracture and calcium intake was also evaluated. Simple linear regressions and fitting plots were used to estimate the correlations between vBMD and calcium intake. Analyses were stratified by sex owing to potential different pathological mechanisms for osteoporosis in women and men [40]. Data with missing BMD or calcium intake were excluded from the data analyses.

The Statistical Analysis System (SAS 9.4 for Windows; SAS Institute Inc., Cary, NC, USA) software was used for all statistical analyses in this study.

## Supplementary Material

Supplementary Figures

Supplementary Tables
