# Supplementary material for: The association of calcium intake with osteoporotic vertebral fractures in a large Chinese cohort
Source: Aging (Albany NY). 2020 Mar 28;12(6):5500–15. doi: 10.18632/aging.102974 (PMC7138559; doi:10.18632/aging.102974)
Supplement: Supplementary Figures [file aging-12-102974-s001..pdf]

SUPPLEMENTARY FIGURES

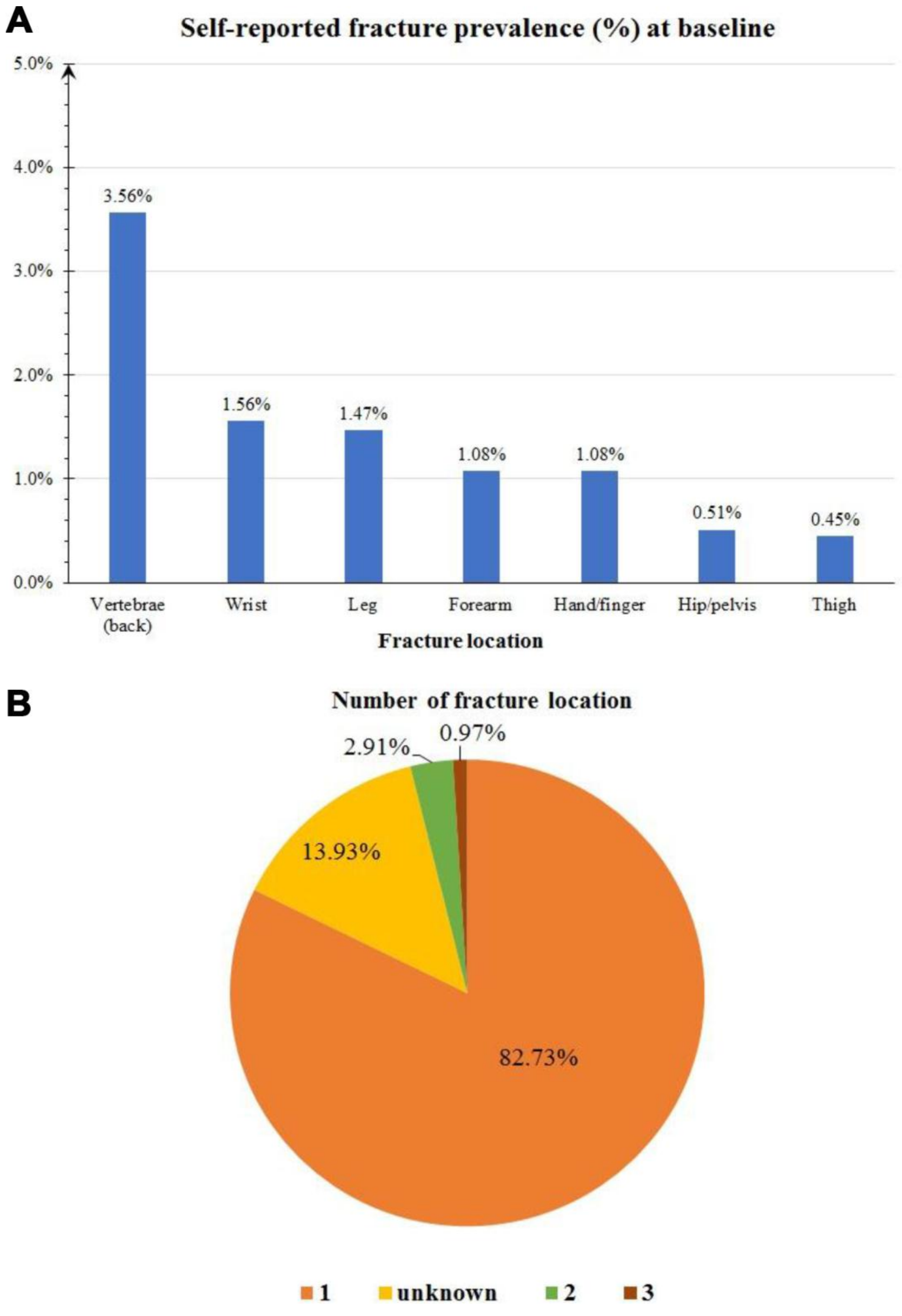

Supplementary Figure 1. (**A** and **B**) Self-reported fracture prevalence and location among Chinese adults aged between 35 and 70 years at baseline.

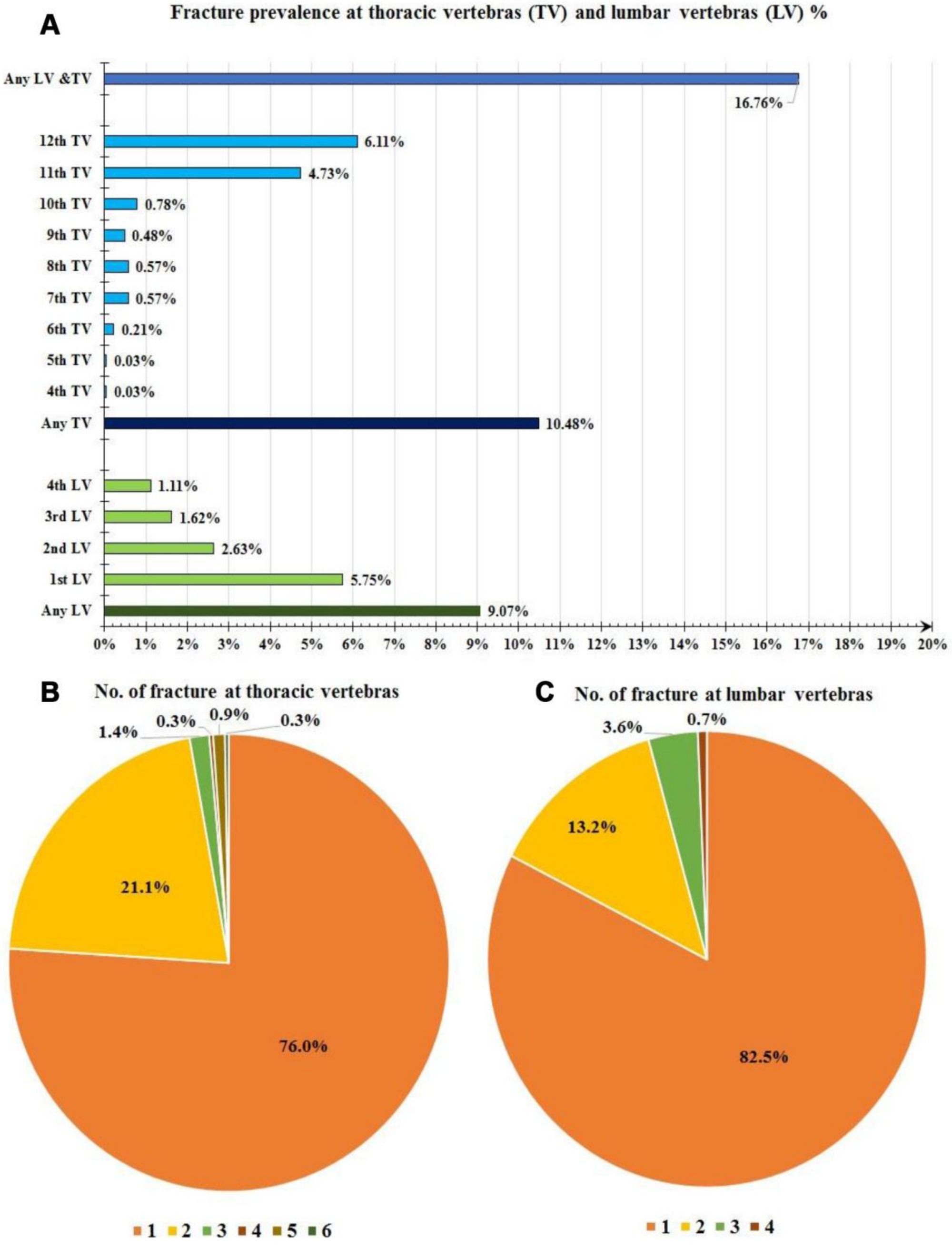

Supplementary Figure 2. (**A** and **B**) Fracture incidence and location at thoracic vertebra (TV) and lumbar vertebra (LV) during cohort follow-up.
