# Supplementary material for: The association of calcium intake with osteoporotic vertebral fractures in a large Chinese cohort
Source: Aging (Albany NY). 2020 Mar 28;12(6):5500–15. doi: 10.18632/aging.102974 (PMC7138559; doi:10.18632/aging.102974)
Supplement: Supplementary Tables [file aging-12-102974-s002..pdf]

SUPPLEMENTARY TABLES

Supplementary Table 1. The CT scanner, scan parameters and recruitment in various centers.

Geographical regions	Center	CT scanner	Scan kVp	Scan mAs	SFOV (mm)	Slice Thickness (mm)
Beijing	Beijing Jishuitan Hospital	Toshiba Aquilion PRIME 80	120	187	500	1
	Beijing Shijingshan Hospital	GE LightSpeed VCT 64	120	150	500	1.25
Liaoning Province	Shenyang 4th Hospital	GE LightSpeed 16	120	150	500	1.25
Sichuan Province	Dayi Hospital	GE Optima CT660 64	120	150	500	1.25
	Huaxi 2nd Hospital	Philips Brilliance 6	120	150	500	2
	Chengdu 2nd People's Hospital	Philips Brilliance 64	120	150	500	1
Jiangsu Province	Changzhou Wujing Hospital	SIEMENS SOMATOM Definition AS+	120	150	500	1
	No1 TCM Hospital	GE Optima CT660 64	120	150	500	1.25
	No2 TCM Hospital	Philips iCT 256	120	238	500	1
Shanxi Province	Taiyuan Central Hospital	Toshiba Aquilion 64	120	75	500	1
Shaanxi Province	Red Cross Hospital of Shaanxi	Philips Ingenuity CT 64	120	150	500	1
Jiangxi Province	Jiangxi Traditional Medicine Hospital	GE Medical Systems Discovery CT750hd 64	120	150	500	1.25

Note: TCM, traditional Chinese medicine; SFOV, standard field of view.

Supplementary Table 2. Sensitivity analyses for vertebral fracture with calcium intake among women by menopausal status[1].

Calcium intake from food	Fracture risk % (n)			Dichotomous odds ratio (95% confidence interval)[2]			Ordinal odds ratio (95% confidence interval)[3]		
	No	Mild	Moderate or severe	Crude	Adjusted[4]	Adjusted[5]	Crude	Adjusted[4]	Adjusted[5]
**Premenopausal**									
Per 100-unit increase	717 (93.2)	42 (5.5)	10 (1.3)	0.92 (0.82, 1.03)	0.92 (0.81, 1.04)	0.92 (0.81, 1.05)	0.92 (0.82, 1.03)	0.92 (0.81, 1.04)	0.93 (0.81, 1.05)
P value				*0.16*	*0.17*	*0.22*	*0.16*	*0.18*	*0.24*
Q1	137 (90.1)	11 (7.2)	4 (2.6)	ref.	ref.	ref.	ref.	ref.	ref.
Q2	160 (93.6)	9 (5.3)	2 (1.2)	0.62 (0.28, 1.40)	0.49 (0.21, 1.14)	0.43 (0.18, 1.02)	0.63 (0.28, 1.41)	0.51 (0.22, 1.19)	0.45 (0.19, 1.07)
Q3	132 (91.7)	11 (7.6)	1 (0.7)	0.82 (0.37, 1.81)	0.58 (0.25, 1.35)	0.51 (0.21, 1.24)	0.83 (0.38, 1.84)	0.63 (0.27, 1.47)	0.58 (0.24, 1.39)
Q4	125 (95.4)	5 (3.8)	1 (0.7)	0.44 (0.16, 1.15)	0.33 (0.12, 0.89)	0.31 (0.11, 0.85)	0.44 (0.17, 1.17)	0.34 (0.12, 0.93)	0.32 (0.11, 0.89)
Q5	163 (95.3)	6 (3.5)	2 (1.2)	0.45 (0.18, 1.08)	0.39 (0.16, 0.95)	0.38 (0.15, 0.98)	0.45 (0.19, 1.09)	0.40 (0.16, 0.99)	0.40 (0.15, 1.03)
P_{trend}				*0.29*	*0.13*	*0.11*	*0.30*	*0.17*	*0.15*
Postmenopausal									
Per 100-unit increase	986 (78.6)	184 (14.7)	84 (6.7)	*0.93 (0.89, 0.99)*	*0.92 (0.87, 0.97)*	*0.94 (0.89, 1.00)*	*0.94 (0.89, 0.99)*	*0.93 (0.87, 0.98)*	*0.94 (0.89, 1.00)*
P value				*0.01*	*<0.01*	*0.03*	*0.02*	*0.01*	*0.06*
Calcium intake quintiles									
Q1	190 (74.8)	43 (16.9)	21 (8.3)	ref.	ref.	ref.	ref.	ref.	ref.

Q2	168 (76.0)	34 (15.4)	19 (8.6)	0.95 (0.63, 1.43)	0.83 (0.54, 1.26)	0.88 (0.57, 1.37)	0.94 (0.62, 1.42)	0.85 (0.55, 1.30)	0.91 (0.58, 1.42)
Q3	187 (77.3)	35 (14.5)	20 (8.3)	0.88 (0.59, 1.33)	0.74 (0.49, 1.12)	0.80 (0.52, 1.23)	0.87 (0.58, 1.32)	0.75 (0.49, 1.14)	0.81 (0.52, 1.26)
Q4	220 (80.9)	38 (14.0)	14 (5.2)	0.69 (0.46, 1.05)	0.54 (0.36, 0.83)	0.60 (0.39, 0.95)	0.70 (0.46, 1.06)	0.57 (0.37, 0.88)	0.64 (0.41, 1.00)
Q5	221 (83.4)	34 (12.8)	10 (3.8)	0.58 (0.38, 0.89)	0.50 (0.32, 0.78)	0.59 (0.37, 0.93)	0.59 (0.39, 0.91)	0.53 (0.34, 0.83)	0.62 (0.39, 0.99)
P_{trend}				*0.08*	*0.01*	*0.09*	*0.10*	*0.03*	*0.17*

[1]Data of menopausal status were obtained via self-reporting at PURE baseline, not at QCT measurement.
[2]Mild, moderate, severe fracture were combined defined as facture prevalent and no fracture.
[3]Three categories were defined as moderate/severe fracture, mild fracture, and no fracture.
[4]Adjusted for age.
[5]Adjusted for age, education level, BMI, waist circumference, tobacco use, alcohol consumption, and physical activities.

Supplementary Table 3. Sensitivity analyses for vertebral fracture with calcium intake among women by age groups.

Calcium intake from food	Fracture risk % (n)			Dichotomous odds ratio (95% confidence interval)[1]			Ordinal odds ratio (95% confidence interval)[2]		
	No	Mild	Moderate or severe	Crude	Adjusted[3]	Adjusted[4]	Crude	Adjusted[3]	Adjusted[4]
**<55 years**									
Per 100-unit increase	505 (95.6)	21 (4.0)	2 (0.4)	0.83 (0.68, 1.02)	0.84 (0.68, 1.03)	0.90 (0.73, 1.10)	0.83 (0.68, 1.02)	0.84 (0.68, 1.03)	0.90 (0.73, 1.10)
P value				*0.08*	*0.09*	*0.30*	*0.08*	*0.08*	*0.29*
Q1	104 (91.2)	9 (7.9)	1 (1.2)	ref.	ref.	ref.	ref.	ref.	ref.
Q2	119 (96.0)	5 (4.0)	0 (0.0)	0.44 (0.14, 1.32)	0.44 (0.15, 1.34)	0.52 (0.17, 1.64)	0.44 (0.15, 1.32)	0.45 (0.15, 1.35)	0.52 (0.17, 1.64)
Q3	92 (93.9)	6 (6.1)	0 (0.0)	0.67 (0.24, 1.93)	0.67 (0.23, 1.94)	0.79 (0.25, 2.54)	0.68 (0.24, 1.94)	0.68 (0.24, 1.96)	0.79 (0.25, 2.55)
Q4	81 (98.8)	1 (1.2)	0 (0.0)	0.13 (0.02, 1.02)	0.13 (0.02, 1.06)	0.17 (0.02, 1.36)	0.13 (0.02, 1.02)	0.13 (0.02, 1.06)	0.17 (0.02, 1.36)
Q5	109 (99.1)	0 (0.0)	1 (1.2)	0.10 (0.01, 0.76)	0.10 (0.01, 0.77)	0.16 (0.02, 1.28)	0.10 (0.01, 0.76)	0.10 (0.01, 0.77)	0.16 (0.02, 1.28)
P_{trend}				*0.07*	*0.08*	*0.23*	*0.07*	*0.08*	*0.22*
**≥55 years**									
Per 100-unit increase	1222 (80.2)	209 (13.7)	92 (6.0)	*0.93 (0.89, 0.98)*	*0.92 (0.87, 0.97)*	*0.94 (0.89, 0.99)*	*0.94 (0.89, 0.99)*	*0.93 (0.88, 0.98)*	*0.94 (0.89, 1.00)*
P value				*0.01*	*<0.01*	*0.02*	*0.01*	*0.01*	*0.04*
Calcium intake quintiles									
Q1	227 (76.4)	46 (15.5)	24 (8.1)	ref.	ref.	ref.	ref.	ref.	ref.
Q2	220 (78.3)	40 (14.2)	21 (7.5)	0.90 (0.61, 1.32)	0.81 (0.54, 1.20)	0.82 (0.54, 1.24)	0.90 (0.61, 1.33)	0.83 (0.56, 1.25)	0.85 (0.56, 1.30)
Q3	230 (78.8)	41 (14.0)	21 (7.2)	0.87 (0.60, 1.28)	0.72 (0.48, 1.07)	0.75 (0.50, 1.14)	0.87 (0.59, 1.29)	0.74 (0.50, 1.11)	0.77 (0.51, 1.18)
Q4	269 (82.5)	42 (12.9)	15 (4.6)	0.68 (0.46, 1.00)	0.53 (0.36, 0.80)	0.57 (0.38, 0.88)	0.69 (0.46, 1.02)	0.57 (0.38, 0.85)	0.60 (0.39, 0.93)
Q5	276 (84.4)	40 (12.2)	11 (3.4)	0.59 (0.39, 0.87)	0.53 (0.35, 0.80)	0.59 (0.39, 0.91)	0.60 (0.40, 0.90)	0.56 (0.37, 0.84)	0.63 (0.41, 0.97)
P_{trend}				*0.05*	*0.01*	*0.06*	*0.08*	*0.02*	*0.12*

[1]Mild, moderate, severe fracture were combined defined as facture prevalent and no fracture.
[2]Three categories were defined as moderate/severe fracture, mild fracture, and no fracture.
[3]Adjusted for age.
[4]Adjusted for age, education level, BMI, waist circumference, tobacco use, alcohol consumption, and physical activities.
